# Comparing the Microvascular Specificity of the 3- and 7-T BOLD Response Using ICA and Susceptibility-Weighted Imaging

**DOI:** 10.3389/fnhum.2013.00474

**Published:** 2013-08-09

**Authors:** Alexander Geißler, Florian Ph. S. Fischmeister, Günther Grabner, Moritz Wurnig, Jakob Rath, Thomas Foki, Eva Matt, Siegfried Trattnig, Roland Beisteiner, Simon Daniel Robinson

**Affiliations:** ^1^Study Group Clinical fMRI, Department of Neurology, Medical University of Vienna, Vienna, Austria; ^2^High Field Magnetic Resonance Imaging Center of Excellence, Medical University of Vienna, Vienna, Austria; ^3^Department of Biomedical Imaging and Image-guided Therapy, High Field Magnetic Resonance Imaging Center of Excellence, Medical University of Vienna, Vienna, Austria

**Keywords:** fMRI, specificity, BOLD, susceptibility-weighted imaging, independent component analysis

## Abstract

In functional MRI it is desirable for the blood-oxygenation level dependent (BOLD) signal to be localized to the tissue containing activated neurons rather than the veins draining that tissue. This study addresses the dependence of the specificity of the BOLD signal – the relative contribution of the BOLD signal arising from tissue compared to venous vessels – on magnetic field strength. To date, studies of specificity have been based on models or indirect measures of BOLD sensitivity such as signal to noise ratio and relaxation rates, and assessment has been made in isolated vein and tissue voxels. The consensus has been that ultra-high field systems not only significantly increase BOLD sensitivity but also specificity, that is, there is a proportionately reduced signal contribution from draining veins. Specificity was not quantified in prior studies, however, due to the difficulty of establishing a reliable network of veins in the activated volume. In this study we use a map of venous vessel networks extracted from 7 T high resolution Susceptibility-Weighted Images to quantify the relative contributions of micro- and macro-vasculature to functional MRI results obtained at 3 and 7 T. High resolution measurements made here minimize the contribution of physiological noise and Independent Component Analysis (ICA) is used to separate activation from technical, physiological, and motion artifacts. ICA also avoids the possibility of timing-dependent bias from different micro- and macro-vasculature responses. We find a significant increase in the number of activated voxels at 7 T in both the veins and the microvasculature – a BOLD sensitivity increase – with the increase in the microvasculature being higher. However, the small increase in sensitivity at 7 T was not significant. For the experimental conditions of this study, our findings do not support the hypothesis of an increased specificity of the BOLD response at ultra-high field.

## Introduction

In functional MRI it is desirable for the blood-oxygenation level dependent (BOLD) signal to be localized, as closely as possible, to the site of neurons activated by a task. Veins draining the capillary bed also give rise to BOLD signal changes, however, leading to a shift in the detected signal away from its origins (Yacoub et al., 2001; Shmuel et al., 2007). The proportion of the BOLD response (quantified either by the number of activated voxels, or mean *Z* value) that arises in tissue to that which comes from the draining veins defines the specificity of the BOLD response. A body of evidence suggests that the relative contribution of draining veins (Menon, 2012) decreases with field strength, leading to the expectation that in ultra-high field functional MRI (fMRI) the measured BOLD signal is better localized to its origin in gray matter (Gati et al., 1997; Ogawa et al., 1998; Yacoub et al., 2001; Duong et al., 2003). These studies are based on numeric models, and measurements examining signal changes and relaxation rate changes in isolated veins and tissue voxels. To date, however, specificity has not been measured with activation statistics or quantified over the whole activated volume, due to difficulty in establishing a reliable network of veins.

Questions as to the exact vascular origin of BOLD signal changes began to be raised soon after the first human fMRI experiments (Bandettini et al., 1992; Kwong et al., 1992; Ogawa et al., 1992). In 1993, Gomiscek et al. indicated that inflow effects originating in large vessels might be a relevant source of the fMRI signal (Gomiscek et al., 1993). Haacke et al. (1994) proceeded to demonstrate that the high signal changes observed in FLASH-based fMRI at 1.5 T were due to large vessels rather than the parenchyma. This finding was supported by experiments in which Stejskal–Tanner gradients, which suppress signal from flowing blood, were included in measurement sequences (Boxerman et al., 1995). The BOLD fMRI signal was reduced by 70–100%, demonstrating that the 1.5-T BOLD fMRI signal originates predominantly from blood in vessels rather than tissue.

Later experiments across the field strengths 0.5, 1.5, and 4.0 T demonstrated that the percentage signal change between rest and activated conditions increases more than linearly with field strength in tissue but less than linearly in vessels (Gati et al., 1997). These findings were extended to 7.0 T, and it was established that the short T2 of blood at high magnetic field was the origin of the reduced vessel contribution at very high field given the relatively long echo time used in fMRI (Yacoub et al., 2001). These studies provided evidence of and an explanation for an increase in the relative specificity of the BOLD signal to gray matter with field strength. The effect has only been measured in isolated vessels identified in T1 and T2 scans, however. The extent to which any changes in the relative contribution of the tissue and vessel signal affects the localization of BOLD signal in a bulk volume of activated tissue is clearly dependent on the distribution of veins in the imaged volume, however. In this study we define maps of venous vessel networks from 7 T Susceptibility-Weighted Images (SWI) (Reichenbach et al., 1997, 1998) to allow the relative contributions of vessel and tissue signal to fMRI results obtained at 3 and 7 T to be quantified. Applying independent component analysis (ICA), rather than a General Linear Model analysis, allows a clean separation of activation from technical, physiological, and motion artifacts, and avoids the possibility of bias to draining vein or microvasculature responses which could have different timing and thereby influence the assessment of specificity.

## Materials and Methods

### Healthy subjects

Twelve healthy, right handed volunteers (eight male, four female, mean age 31.6 years, age range from 23 to 45) participated in the study, which was approved by the Ethics Committee of the Medical University of Vienna, with written informed consent.

### Task design and procedure

A hand motor task was chosen because it elicits a strong and reproducible BOLD response which is localized in a well circumscribed region. Volunteers were instructed to perform repetitive opening and closing of the right hand at 1 Hz. Auditory start and stop commands were computer-generated and communicated via the scanner intercom system. The sequence of timed commands was executed with the software Presentation (Neurobehavioral Systems, Albany, CA, USA) and was triggered by the MRI scanner. All subjects performed a simple blocked design consisting of four movement and five rest periods of 20 s each, with two runs at each field strength.

### Data acquisition

All subjects were examined with both a 3-T Siemens MAGNETOM TIM TRIO scanner and a 7-T Siemens MAGNETOM scanner (Siemens Medical, Erlangen, Germany). A 32 channel head coil was used on both systems (on 3 T, manufactured by Siemens Medical, on 7 T, manufactured by Nova Medical, Wilmington, MA, USA).

Functional data were acquired on both systems with high resolution 2D single shot gradient-echo (GE) EPI, with slices aligned parallel to the AC-PC plane and whole brain coverage. To ensure that results obtained here are relevant to fMRI in general practice, we chose to assess specificity using the echo time which, for each field strength, provides the maximum BOLD sensitivity (approximately equal to T2* in gray matter; Deichmann et al., 2002). Protocols used at 3 and 7 T were also independently optimized according to specific absorption rate (SAR) constraints, the requirement of whole brain coverage and other recommendations in the literature (Triantafyllou et al., 2005; Robinson et al., 2008; Speck et al., 2008; van der Zwaag et al., 2009).

At both field strengths, GE-EPI was acquired with a square field of view (FOV) of 220 mm, in-plane matrix size 220 × 220, with slice thickness of 2 mm and 20% gap (i.e., 1 mm × 1 mm × 2.4 mm voxels), with 73 repetitions, a repetition time (TR) of 3000 ms, fat suppression with a chemical shift selective saturation pulse prior to every slice, 6/8 partial Fourier factor (omitting the first 25% of k-space phase-encoding lines), and parallel imaging with a GRAPPA-iPAT factor of 4. This relatively high GRAPPA factor was required to achieve the desired echo times with these high resolution acquisitions. At 3 T, 37 slices were acquired with TE = 35 ms, a receiver bandwidth per pixel (BW) of 1082 Hz, flip angle (FA) of 90°. At 7 T, 44 slices were acquired with TE = 22 ms, BW = 990 Hz, FA = 75°. As these echo times are different between the two field strengths (35 ms for 3 T, 22 ms for 7 T) we also performed an additional comparison of specificity with a single subject (subject 8) using runs with both echo times – 35 and 22 ms – at both field strengths, to assess to what extent specificity findings are echo-time dependent. A total of four additional motor runs – two with 35 ms and two with 22 ms – were measured at each field strength for subject 8 only.

High resolution, fully flow compensated T2*-weighted 3D GE images were acquired at 7 T for SWI. The acquisition matrix size was 704 × 704 × 96 voxel, with a FOV of 220 mm, leading to 0.3125 mm × 0.3125 mm × 1.2 mm, TE/TR = 11.9/28 ms, FA = 15°, with BW = 163 Hz/px, and an acquisition time of 13 min 20 s.

### Data processing

#### Functional data analysis

Echo planar images were motion corrected using MCFLIRT (Jenkinson et al., 2002) from Version 5.0.1 of the FSL software package (Smith et al., 2004), with all volumes registered to the first volume of the first functional experiment (3 and 7 T separately). ICA was performed in this native EPI space with “MELODIC” (Beckmann and Smith, 2004) for each subject with no smoothing applied. MELODIC was run in multi-session tensorial mode (TICA) without skull stripping but with the brain volumes of interest (VOI) as a confinement. The mean bias-corrected EPI was used as a background image for functional overlays. The threshold for the mixture model-based inference was 0.5 (the default) and the model order, or number of components into which the data is split – was determined automatically using Laplacian estimation.

To identify which voxels overlay veins and which tissue, functional data were registered to SWI space in a number of linear registration steps, with increasing number of degrees of freedom as the quality of the result improved, followed by non-linear registration. Registration was performed using the mean EPI and magnitude SWI image, both of which were skull stripped with BET2 (Jenkinson et al., 2002), with subject-specific fractional intensity thresholds and bias-corrected with FSL’s “FAST” package. Magnitude SWI were additionally denoised with FSL’s non-linear noise reduction tool “SUSAN” and intensity-normalized to a value of 1000. A binary brain mask was generated from this image (by setting all non-zero values to 1). This was for used with FNIRT and for the generation of venous vessel maps (see “Generation of Venous Vessel Maps”).

For 7 T EPI, the first step was linear registration of mean skull stripped, bias-corrected EPI to 7 T SWI using FSL’s “FLIRT” (Jenkinson et al., 2002) with correlation ratio as the cost function and 7 degrees of freedom (three translational, three rotational, and global rescaling). The output matrix of this transformation was used as a starting point for a second execution of FLIRT (again to SWI), using mutual information as the cost function and 12 degrees of freedom. The final registration step was a non-linear transformation of the output of the linear transformations to SWI using “FNIRT.” In the light of the sequence-dependent intensity disparity between EPI and SWI, the local non-linear intensity model was used for FNIRT.

For 3 T EPI, registration steps were as described for 7 T above, other than that they were preceded by the addition step of linear registration to the 7-T EPI using FLIRT with 7 degrees of freedom. The normalized correlation ratio was used as the cost function for all linear registration stages of 3 T data. Normalized correlation ratio is usually used for intermodal registration, but provided the best results in this application due to the contrast differences between 3 and 7 T data. The global non-linear intensity model was used for FNIRT.

This multi-step registration procedure was found to provide accurate registration for all subjects. For both 3 and 7 T fMRI data all transformation steps, both linear and non-linear, were combined to define the transformation from EPI to 7 T SWI. The merged transformations were finally applied to ICA maps. This approach ensured the equal treatment of 3 and 7 T functional data – of a single transformation with one resampling step, vital because every applied transformation causes some smoothing of the data.

#### Generation of venous vessel maps

**Figure 1:**
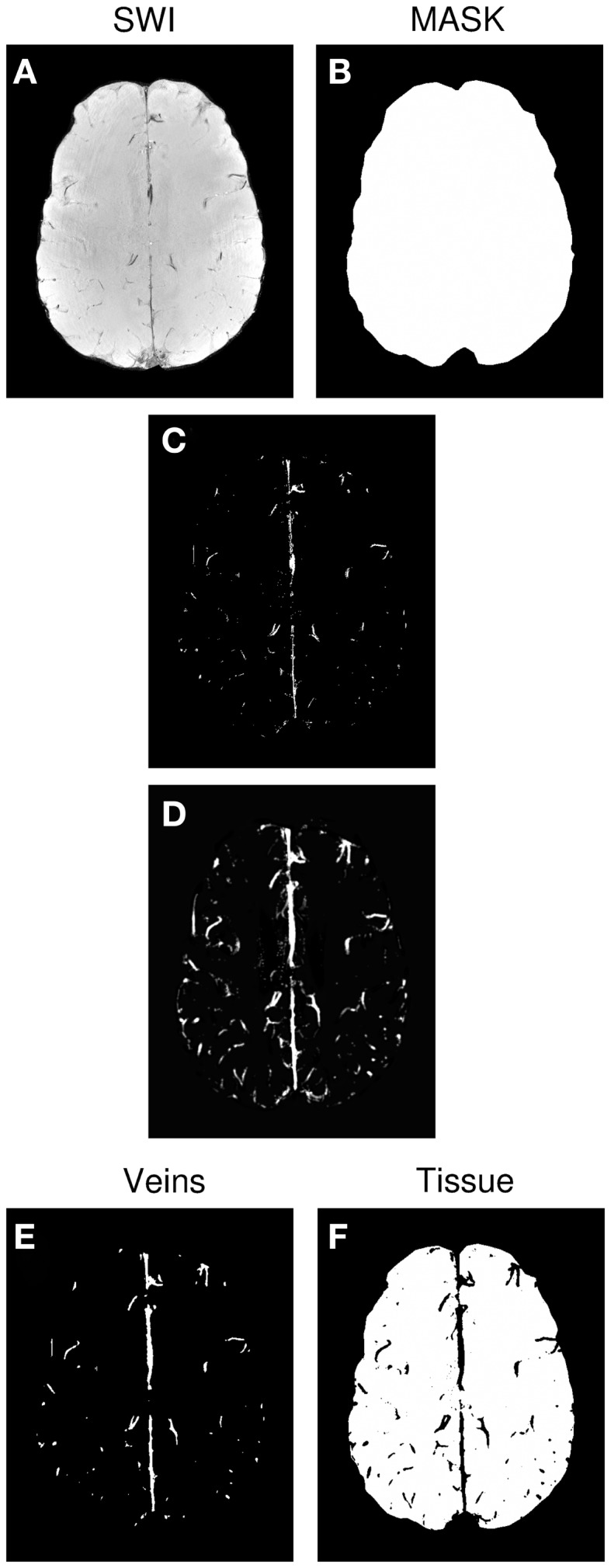
**Generation of the venous vessel maps**. **(A)** SWI. **(B)** Brain mask. **(C)** Preliminary vein mask, achieved by applying threshold “T” to SWI. **(D)** Smoothed version of preliminary vein mask. **(E)** Final vein mask derived from **(D)**. **(F)** Tissue mask derived from**(D)**.

Vessel maps were generated semi-automatically from SWI magnitude images using MATLAB (MathWorks, Inc., Natick, MA, USA). Steps are illustrated in Figure 1, and were as follows. For each subject’s magnitude SWI (Figure 1A), a threshold “T” was determined for the whole volume by hand, below which images were classified as consisting of veins or background signal. Voxels whose value was below T and which were inside a BET mask of the brain (Figure 1B) were set to 1, and all other voxels were set to 0 (Figure 1C). This preliminary vein mask was smoothed using the “smoothn” MATLAB function with the smoothing parameter S of 1 (Garcia, 2009) (Figure 1D). A binary vein map was created by assigning the value of 1 to voxels in the smoothed preliminary mask (Figure 1D) which exceeded a value of 0.3 (yielding Figure 1E). The vein mask was compared by visual inspection with the SWI for the verisimilitude of the vessels identified, and the threshold “T” modified, if necessary. A “tissue” mask was assigned the value of one where values in the smoothed preliminary mask (Figure 1D) were below 0.15 (yielding Figure 1F).

The process of thresholding, smoothing, and thresholding a second time removed isolated voxels and bridged gaps in vessels (compare Figures 1C,E). Using different thresholds for the vein and tissue masks (0.15 and 0.3 respectively) led to a cleaner allocation of voxels to the vein and tissue categories. fMRI activation was classified as being in a vein if it coincided with the vein mask and being in the microvasculature if it was in the tissue mask. Voxels in the zone between the two were not considered in further analysis.

#### Statistical analysis

To assess effects related to the two field strengths under controlled conditions, anatomical VOIs were located within the primary hand motor area. VOI’s were manually defined for each subject by an experienced fMRI expert (RB) and comprised the neurophysiological representation of the human hand area, i.e., the knob structure. The individual VOI was the same for both field strengths. The following values were calculated for both veins and microvasculature: (1) number of activated voxels (alternative hypothesis test at a Gaussian mixture modeling threshold of *p* > 0.5; Beckmann and Smith, 2004), (2) percent activated voxels, (3) mean *Z* value, and (4) ratio of the mean *Z* values in the microvasculature/veins for all voxels within the anatomical VOI. Both the number of activated voxels as well as the mean *t*-values of those voxels was assessed with a Student’s paired two-tailed *t*-test carried out in Microsoft Excel 2007 (Redmond, Washington, MA, USA). A decreased vascular contribution to the BOLD signal is to be expected at 7 T due to the short T2 of blood. As specificity effects can be expected to be echo-time dependent, data at the same echo times (22 and 35 ms) were likewise compared.

## Results

**Figure 2:**
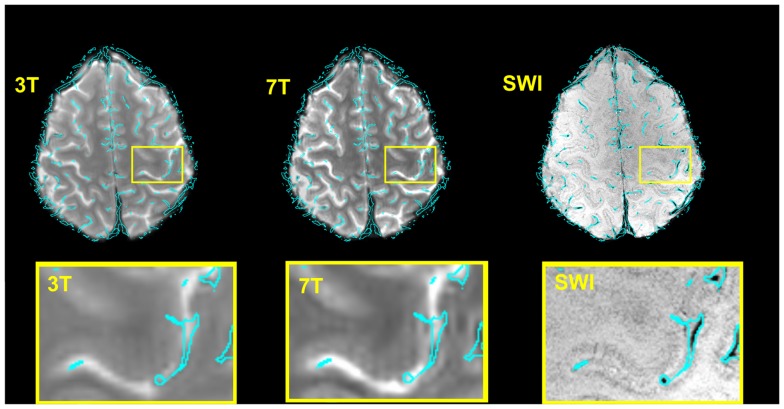
**Verification of the accuracy of the normalization 7 T → SWI space and 3 → 7 T (SWI) space for a typical subject and illustration of the corresponding vein map**. For the illustration only, the boundaries of the veins (rather than the vein masks themselves) are shown, overlaid in cyan. These were generated with the contour function of CorelDraw (Corel Corporation, Ottawa, ON, Canada). Bottom row: zoomed depiction of the hand area.

Figure 2 illustrates, for a single subject, both the accuracy of the image registration and the appearance of the vein maps, the outline of which are overlaid (in cyan) on sample 3 and 7 T EPI volumes, and SWI. Some identified veins appear to lie outside the brain. A proportion of these are genuine veins on the surface of the brain, beyond the cortical surface, which appear further outside the brain due to partial volume effect over slices. In EPI there is also strong T2* dephasing of signal from the periosteal and meningeal dural layers, which makes the brain appears slightly smaller than the skull-stripped SWI, enhancing the impression that these veins lie further outside the brain. Any errors in the vein maps outside the anatomically defined VOIs in the primary motor cortex do not affect our results, as analysis was constrained to those VOIs.

**Figure 3:**
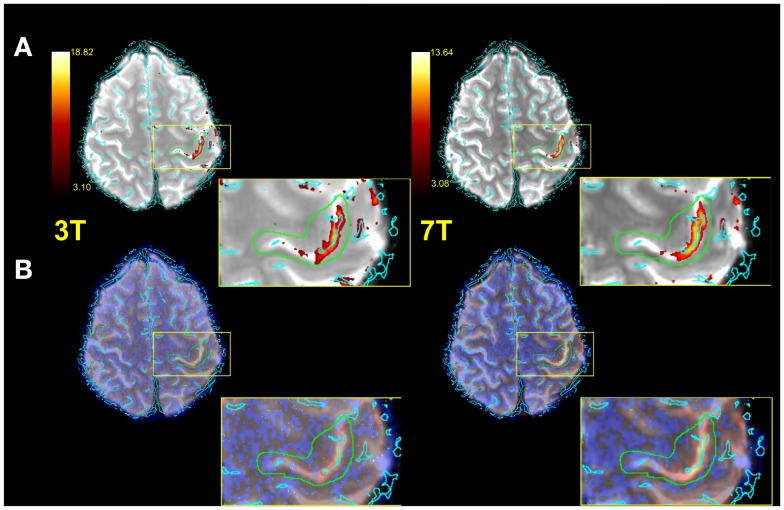
**Exemplified single subject illustration (radiological convention)**. **(A)** Functional image with vessels (cyan) thresholded IC map and anatomical VOI (green) overlaid. **(B)** As in **(A)**, but with no thresholding applied to IC map. A zoomed representation of the target area is also illustrated.

Figure 3 illustrates typical functional results within the predefined anatomical VOI (green) for a single subject. Row A shows voxels above threshold (determined via the Gaussian mixture modeling approach described in the see “Statistical Analysis”), row B all functional voxels. The zoomed depiction clarifies the situation inside the target area. Both the number of voxels above threshold as well as the mean *Z* values of all voxels in the VOI were assessed for each subject (see Table 1).

**Table 1:** **Individual subject results showing the number of activated voxels and mean *Z* values in regions identified as vessels and microvasculature**.

3 T	Subject	Number of activated voxels in VOI			Mean *Z* over all voxel in VOI		
		Vessels	μvasc	% In μvasc	Vessel	μvasc	*Z* μv/*Z* vessel
	1	1521	17,367	91.9	2.1	1.3	0.61
	2	1323	6982	84.1	2.6	1.1	0.41
	3	1345	8058	85.7	2.2	1.0	0.48
	4	1209	5056	80.7	1.9	1.0	0.53
	5	1926	4720	71.0	2.1	0.7	0.33
	6	2339	8856	79.1	2.8	1.4	0.50
	7	1314	6746	83.7	1.9	0.8	0.44
	8	2083	8097	79.5	3.3	1.2	0.37
	9	1655	7075	81.0	1.8	0.8	0.44
	10	2124	13,940	86.8	3.6	1.5	0.41
	11	1026	7732	88.3	1.8	1.0	0.54
	12	2094	14,617	87.5	2.2	1.0	0.45
	**Mean**	**1670**	**9100**	**82.9**	**2.4**	**1.1**	**0.45**
	**SD**	**430**	**4000**	**7.5**	**0.6**	**0.2**	**0.08**
**7 T**	1	1986	25,895	68.0	2.8	1.8	0.64
	2	1366	9849	92.9	3.3	1.8	0.53
	3	1840	10,869	87.8	3.4	1.7	0.50
	4	1269	5598	85.5	2.3	1.4	0.59
	5	2422	11,218	81.5	3.0	1.5	0.49
	6	2392	11,206	82.2	2.9	1.6	0.56
	7	1664	9019	82.4	2.3	1.0	0.45
	8	2343	8490	84.4	3.4	1.3	0.38
	9	1804	5316	78.4	2.3	0.9	0.41
	10	2339	20,568	74.7	4.9	2.0	0.40
	11	2213	16,217	89.8	3.9	1.8	0.45
	12	2780	19,780	88.0	4.5	1.8	0.40
	**Mean**.	**2030**	**12,800**	**84.3**	**3.2**	**1.5**	**0.47**
	**SD**	**460**	**6400**	**5.6**	**0.8**	**0.3**	**0.08**
**3/7 T stats**	***t*-Test**	0.0025*	0.0025*	**n.s**.	0.0017*	7.33E−05*	**n.s**.

All values are investigated within neuroanatomically defined VOIs. *Indicate statistically significant differences between 3 and 7 T, and “n.s.,” indicates a non-significant result.

On average, 21% more voxels were above threshold in veins at 7 T than at 3 T and 42% more voxels were above threshold in the microvasculature at 7 T than at 3 T (see Table 2). These increases in BOLD sensitivity with field strength in both veins and the microvasculature were statistically significant in student’s two-tailed *t*-tests assessed at *p* < 0.05. The proportion of activated voxels in the microvasculature to the total did not differ significantly between the two field strengths, however, indicating no increase in specificity.

**Table 2:** **Summary sensitivity and specificity results extracted from Table 1**.

**7/3 T SENSITIVITY**
*N* vessels	1.21 (0.31)
*N* μvasc	1.42 (0.44)
*Z* vessels	1.41 (0.35)
*Z* μvasc	1.48 (0.32)
**7/3 T SPECIFICITY**
% In μvasc	1.01 (0.13)
*Z* μv/*Z* vessel	1.07 (0.17)

*Values in brackets are standard deviations on the mean*.

Mean *Z* values were significantly higher in the 7-T results in both the vessels and the microvasculature. In veins, the increase was 41%, in the microvasculature it was 48%. The increase in the ratio of mean *Z* values in the microvasculature to veins with field strength was small and not statistically significant, indicating no increase in specificity. The same finding, of no substantial increase in specificity, held when the assessment was carried out at the same echo time (Table 3).

**Table 3:** **A comparison of functional specificities measured at two the same echo times at 3 and 7 T in one subject (subject 8; mean over two runs)**.

3 T	Echo time (ms)	Number of activated voxels in VOI			Mean *Z* over all voxel in VOI		
		Vessels	μvasc		Vessel	μvasc	Ratio
				
			μvasc	% In μvasc	*Z* mean	*Z* mean	*Z* μv/*Z* vessel
	22	1735	10,608	85.94	2.55	1.41	0.55
	35	2028	13,004	86.51	2.88	1.63	0.57
7 T	22	3561	28,622	88.94	8.15	3.38	0.41
	35	3630	31,608	89.70	7.81	3.71	0.48

*All values are investigated within neuroanatomically defined VOIs. There is no substantial increase in specificity, measured via percentage of activated voxels in the microvasculature, or the ratio of mean Z values in the microvasculature to that in vessels, with field strength, even if the same echo times are used at 3 and 7 T*.

## Discussion

The field strength dependence of the specificity of the BOLD response has been studied using high resolution fMRI at 3 and 7 T with a hand task. ICA was used to identify task-related activation in order to obviate possible bias of a model-based analysis to either the vascular or microvascular response, as these could be subject to different latencies. Activation maps were meticulously normalized to the space of vessel maps derived from high resolution 7 T SWI scans using state-of-the-art non-linear image registration. The results of this analysis allowed both the relative sensitivity and the relative specificity of the BOLD response at 3 and 7 T to be assessed.

There was significant increase in the number of activated voxels at 7 T in both the veins and the microvasculature, with the increase in the microvasculature being higher. The increase in both tissue classes confirms the increase in BOLD sensitivity of 7 T fMRI observed in other studies (Triantafyllou et al., 2005; van der Zwaag et al., 2009; Beisteiner et al., 2011). While the fact that there was a larger increase in the number of voxels activated in the microvasculature might suggest an increase in microvascular specificity at 7 T, this tendency was non-significant due to high variance over subjects. Findings were the same for mean *Z* values. There were significant increases, of ∼40%, in *Z* values in both the veins and tissue in 7 T results compared to 3 T results. Again, the increase was consistently higher in tissue, but not significantly so. The obvious conclusion, that BOLD specificity is not significantly higher at 7 T than at 3 T could be affected by our choice of echo times, which was different (and near optimum) for each field strength (35 ms for 3 T and 22 ms for 7 T) (Yacoub et al., 2001; Robinson et al., 2004). We tested the generality of our conclusion, however, by performing additional measurements at both 22 and 35 ms at each field strength. Although there was a small difference in all Z values between echo times the ratio *Z* μvasc/*Z* vessel did not differ substantially. We therefore conclude that while changing echo-time (unsurprisingly) influences BOLD sensitivity, it did not, to a measurable degree, affect specificity.

Our primary hypothesis in this study was that the increase in sensitivity would be larger in tissue than veins, demonstrating an increase in specificity and indicating that improved localization of the BOLD signal is to be expected at ultra-high field. This could not be confirmed, in apparent contradiction of prior work. For instances, Gati et al. (1997) predicted larger signal increases in tissue than veins in the visual cortex at 0.5, 1.5, and 4.0 T on the basis of measurement of SNR, ΔR2*, and R2* at these field strengths. Yacoub et al. (2001) extended Gati et al.’s findings relating to ΔR2* and R2* from 4 to 7 T, but likewise based their predictions on increasing signal changes compared to relatively constant noise. The authors assumed that thermal noise “will ultimately dominate the noise term” – i.e., that when physiological noise is better understood and imaging systems are more developed, physiological noise will be reduced to below the level of thermal noise. Despite progress on this front (e.g., Boyacioglu and Barth, 2012), this point has not yet been reached. One source of physiological noise is pulsatory blood flow. In conventional EPI at least, the signal changes related to pulsatory flow (which typically have a frequency of 1–1.5 Hz) are undersampled with TRs of ∼0.5 Hz, and other physiological noise sources cannot be comprehensively removed, meaning that physiological noise is still at least as large as thermal noise at 7 T (Triantafyllou et al., 2005). The relatively high resolution measurements we made here with full brain coverage and accelerated imaging go as far as possible to reducing the relative contribution of physiological noise to the total (physiological plus thermal noise), and thereby yield the best specificity possible. A relatively high GRAPPA factor of 4 was used to achieve the desired echo times with these high resolution acquisitions. While the use of high parallel imaging factors increases g-factor noise and reduces BOLD sensitivity, the BOLD sensitivity in this study was sufficient to detect activation in the primary motor cortices of all subject. This GRAPPA factor is not expected to have any influence on specificity.

Fully automatic identification of vessels from SWI scans is a complex process and the subject of considerable research effort as a separate field (see, e.g., Frangi et al., 1998). Our attempts to apply a leading existing approach (Kroon, 2009) in this study led to imperfect detection of vessels and false positive vessel detection and enlarged vessels, and motivated the development of our own method. While our simple magnitude threshold-based approach performed much better than existing methods with this data it is also subject to shortcomings. Firstly, a threshold needs to be set to determine where veins end (how broad a vein mask is for a given appearance in SWI) and where tissue begins. Our vein maps were defined quite conservatively and a margin was left before defining voxels as belonging to tissue. In this way, we minimized the influence of this border zone between vein and tissue on our specificity results. Veins have low signal in SWI, but so does CSF and the interhemispheric fissure, so these regions are erroneously included in the vein mask. These errors did not influence results obtained here, as all analysis was performed within a VOI for the primary motor cortex which excluded these problems, but would lead to errors if applied uncritically in other studies.

It should be noted that our finding of no demonstrable increase in specificity at 7 T compared to 3 T are constrained to the motor system. A motor paradigm was chosen due to its robustness and our group’s interest in precise localization of motor function in the context of presurgical planning (Beisteiner et al., 1995, 2001). Future work could involve extending this examination to the visual system, which would afford more direct comparison with prior studies, although specificity findings should be independent of the region studied. Finally, these findings are constrained to the measurement sequence and methods applied at both field strengths. Future developments in fast imaging may allow both the sensitivity and specificity of ultra-high field fMRI to be increased (Poser et al., 2013).

In summary, this study, comparing high resolution fMRI of the motor system at 3 and 7 T, does not confirm a significant increase in the specificity of the BOLD response at ultra-high field.

## Conflict of Interest Statement

The authors declare that the research was conducted in the absence of any commercial or financial relationships that could be construed as a potential conflict of interest.
